# Democratizing Global Health Care Through Scalable Emergent (Beyond the Mobile) Wireless Technologies

**DOI:** 10.2196/31079

**Published:** 2022-02-11

**Authors:** Graham B Jones, Andrew Bryant, Justin Wright

**Affiliations:** 1 Technical Research and Development Novartis Pharmaceuticals East Hanover, NJ United States; 2 Technical Research and Development Novartis AG Basel Switzerland; 3 Global Drug Development Connected Health Novartis Pharmaceuticals East Hanover, NJ United States

**Keywords:** LPWAN, wireless communication, global health, mobile technology, mHealth, wireless, cloud-based, personalization, cost, security, convenience, IoT, Internet of Things, global health

## Abstract

Advances in mobile phone technologies coupled with the availability of modern wireless networks are beginning to have a marked impact on digital health through the growing array of apps and connected devices. That said, limited deployment outside of developed nations will require additional approaches to collectively reach the 8 billion people on earth. Another consideration for development of digital health centered around mobile devices lies in the need for pairing steps, firmware updates, and a variety of user inputs, which can increase friction for the patient. An alternate, so-called Beyond the Mobile approach where medicaments, devices, and health services communicate directly to the cloud offers an attractive means to expand and fully realize our connected health utopia. In addition to offering highly personalized experiences, such approaches could address cost, security, and convenience concerns associated with smartphone-based systems, translating to improved engagement and adherence rates among patients. Furthermore, connecting these Internet of Medical Things instruments through next-generation networks offers the potential to reach patients with acute needs in nonurban regions of developing nations. Herein, we outline how deployment of Beyond the Mobile technologies through low-power wide-area networks could offer a scalable means to democratize digital health and contribute to improved patient outcomes globally.

## Introduction

The 1962 song entitled *Return to Sender* by Elvis Presley harkens back to an age when people communicated through letter writing, and the postal service was the principal artery of our data distribution network [1]. Lest we forget, there are several features of that communication method that are noteworthy and can still inspire innovation within our modern world of telecommunications. First, the cost of the communication was borne upfront by the originator, by using either a stamp or franking imprint. The recipient’s address was unambiguous, and the act of opening the communication was personal and often ethereal. There is also the option to respond to the sender at no cost with a return letter through an enclosed “self-stamped” return envelope. The modern equivalents are of course electronic, offering near-unlimited speed, scale, and customization, though this comes at a cost. The first and most obvious is a lack of personal touch. Although this may have little consequence in most circumstances, in the case of health care, we must be guided by the wishes, needs, and proclivities of a potentially unwell person whose perspectives may differ from those of a well-intentioned app developer. Second, modern communications rely on mobile devices that can be expensive (capital expenditure), have limited shelf life (depreciation), and are coupled through networks that may require monthly subscriptions (operational expenditure). In addition to these financial burdens, the devices rely on availability of increasingly complex wired and wireless networks that have colored our expectations dramatically. Whereas a delay of one day for receipt of a letter might have been a source of irritation in days gone by, a lack of internet connectivity for mere minutes now has the potential to drive consumers into a state of frenzied panic. More problematically, the market push for ever-increasing device capabilities and networks to support them has implications for global health. There is clear evidence of a digital divide emerging between affluent and developing nations, yet the push toward digital health has the potential to impact the health care of some of the most vulnerable citizens on the planet [2]. We can all recall the frustrations of having to upgrade operating systems on our personal computers to keep pace with the visions and edicts of software developers. In the case of health care, however, such scenarios are less tenable, requiring us to plan carefully and thoughtfully how we embrace this opportunity. We advocate that the correct combination of digital services coupled with personal actions and experiences may be the solution to this conundrum and outline a case herein.

## Beyond the Mobile

**Figure 1:**
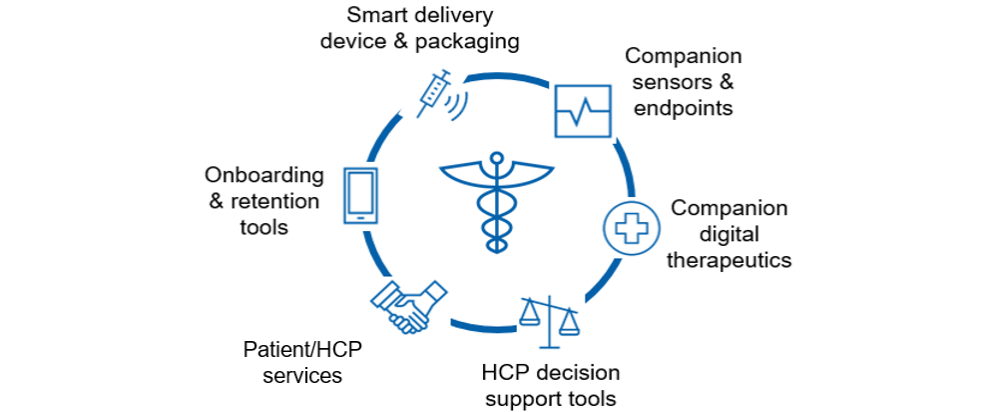
Functional components of a modern Connected Health ecosystem. HCP: health care provider.

The promise of digitally enabled health care is being realized at an incredible pace [3]. Advances in device and broadband network technology are revolutionizing how we capture, store, and access health care data. The range of possibilities is ever expanding, impacting patients, health care providers, and payers with the establishment of Connected Health ecosystems (Figure 1). Consumer interest has been accelerated by the availability of apps on smartphones, allowing patients to access and visualize health-related data and make decisions on aspects of their health care [4].

It is estimated there are approximately 3 billion smartphone users globally and over 318,000 health-related apps available for personal use [5]. That said, the highest concentration is found in developed, affluent nations and their urbanized regions, reflecting demographic trends and mirroring geographic access to quality health care services [6]. This has even led to categorization of a subclass of “fit-rich” individuals who also possess smart monitoring devices (eg, wrist-worn wearables), have ready access to a spectrum of preventative through palliative health care services, and actively engage in digital health programs [7]. Despite the seemingly limitless opportunities, the impact on health outcomes at the population level has been smaller than initially anticipated and device-related limitations have been cited as a likely culprit. Several surveys underscore patients’ diminished enthusiasm for devices over time, including app burnout, with many using features less than 3 times [8]. This presents a conundrum for the connected health community at large. Given the smartphone-centric culture of Generation X, Millennials, and Generation Z, it is logical to assume that connected health products represent an enormous opportunity, and this will likely be the case for a large segment of the health care system. However, at the population level, there are a number of inherent hurdles and limitations that need to be considered a priori for patients to use the smartphone as the central hub in their health care nexus. Cost is a factor, with premium devices exceeding US $1000, and data plans of up to US $100/month. In the United States, the average user changes their smartphone approximately every 24 months, often amortizing sunk cost into a newer model [9]. Among limitations of the base unit, battery life is often a limiting factor, with devices typically requiring daily charging despite continual advances in lithium-ion technologies. This problem is even worse for some wrist-worn systems (eg, the Apple Watch), as their need for regular recharging has been a limiting factor in clinical trials, as on-body data cannot be gathered while the device is charging [10]. The situation becomes even more complex when the smartphone is paired with additional devices—for example, when a smartphone is communicating with a drug substance supplied in a smart package, allowing patients to obtain instructions for use via wireless (near-field or Bluetooth) communication. Another example is a connected drug delivery device such as a smart autoinjector or inhaler. In both cases, in addition to the installation of a customized app, a pairing process needs to be conducted, which can be a source of friction, and requires the smartphone to be in close proximity, with sufficient battery life and wireless connectivity. Longer term, the need for firmware and app updates is likely, requiring a degree of technical knowledge, and there may be potential interoperability limitations if contemplating switching operating systems (eg, from iOS to Android). On top of this, omnipresent security concerns arise, both for the operation of the device and the data it collects then distributes. Though the growth and use of health-related apps on smartphones seems likely to continue unabated, there are merits to using alternate and transparent approaches to connect devices, which has led to the emergence of Beyond the Mobile (BTM) as a viable proposition [11].

**Figure 2:**
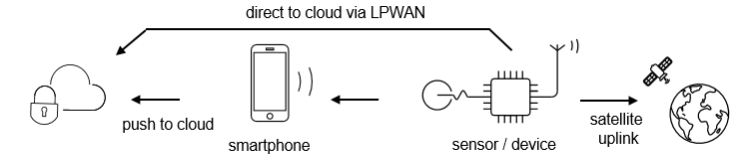
Uploading data from IoMT devices directly or via smartphone. IoMT: Internet of Medical Things; LPWAN: low-power wide-area networks.

In this scenario, a sensor on a wireless device communicates directly to the cloud through a low-power wide area network (LPWAN) or through a satellite uplink without the need for the smartphone-based intermediate step (Figure 2). As just a few examples, a smart pill dispenser could confirm when a patient administers a drug through activation of a container cap sensor, a motion sensor might alert a caregiver to a patient’s sudden fall, or a wrist-worn cardiac monitor might signal an irregular event in need of further scrutiny (eg, arrythmia). Though it is equally viable to capture this information via a smartphone, there are several advantages to a BTM approach. For example, the sensing device could be configured to work “out of the box” with minimal setup and no requirement for a separate device (and thus no need for downloading apps and updates, pairing, etc). In addition, it could be personalized to a user profile up front prior to shipping. Data could be transmitted near instantly via an appropriate network (vide infra) at no cost to the patient, with lessened security concerns as data are not stored on an uncontrolled system, nor transmitted over the open internet.

Additionally, validation of such a device would be less demanding than a smartphone-paired version, as the likelihood of errors from software of unknown provenance is reduced. If debugging was proven necessary, those updates would be confined to the device itself and would not be compounded by issues relating to the smartphone and its various systems. Clearly, there are myriad benefits to such an approach, and the impact of reduced friction for the patient is noteworthy. It is well recognized that to evaluate the true impact of connected health on real-world outcomes, acquiring quality data longitudinally is key, and low friction/passive monitoring approaches are beneficial [12]. This in part has led to the development of wide arrays of newer Internet of Medical Things (IoMT) devices (eg, voice assistants), which can gather data with minimal user inputs [13].

Using BTM approaches to connected care has even deeper ramifications when considering population health at a global scale. Leaving aside limitations of smartphones themselves, their use depends on the availability of cellular networks on which the devices operate. It is estimated that nearly half of the world’s population does not have access to the internet and an even greater percentage does not have access to broadband (cellular or Global System for Mobile communication) networks [14]. The problem is exacerbated in developing nations—whereas some 73% of North Americans have mobile connectivity, the lower proportions of mobile connectivity in Latin America (50%), the Middle East and North Africa (38%), South Asia (28%), and sub-Saharan Africa (21%) highlight the gaps that exist [15]. Although the deployment of 3G, 4G, and now 5G networks continues apace, the economic considerations involved mean that many areas, particularly rural, will remain cold spots for the foreseeable future. In a move to address this infrastructure gap, low-energy long-range wireless technologies are being developed and evaluated, leading to LPWANs [16]. Coupling to these networks through low-energy devices offers a very real and scalable option for impacting connected health in developing nations.

## Long-range Wide Area Networks

First developed by Cycleo in France, LoRa (from “long range”) represents a long-range, low-power wireless protocol that can be deployed through LPWANs [17]. Subsequently acquired by Semtech and incorporated into a global nonprofit, LoRa Alliance, the system employs sub-gigahertz radio frequency bands (principally 433-923 MHz) to allow for long-range data transmission (>15 km rurally) using low power and transfer rates of up to 30 kbit/s [17]. Long-range wide area networks (LoRaWAN) have multiple attributes, which include the following: (1) multikilometer range capacity, (2) low power use through micro batteries, (3) low entry and operating costs (capital expenditure approximately US $20, operational expenditure negligible), (4) low bandwidth requirements (250 bit/s to 11 kbit/s), (5) security available through 128-bit encryption, and (6) near limitless geographic coverage of gateways. There are limitations including low uplink/downlink speeds, favoring methods that parse data into appropriate packets for transmission with intervals, as well as edge computing methods to preanalyze data. Perhaps more importantly in this context, however, the technology uses unlicensed radio bands, and the network technology is relatively simple and affordable to access. The low cost of implementation and lack of formal network licensing leads to the potential for community-driven solutions.

New chipsets are continually evolving, with ever increasing transmission capacity coupled with decreasing size and power consumption which, when augmented with geolocation capability, has resulted in LoRaWAN fast becoming a connection method of interest for Internet of Things devices in remote areas. Following development in Europe, LoRaWAN has been deployed in over 100 countries, which includes many in Asia, Africa, and Latin America [17]. One of the most ambitious deployments to date was undertaken by Tata Communications in India, with a goal to reach 400 million people in 2000 communities [18]. Based on promising data, numerous developing nations are now in the process of assessing how to integrate autonomous LoRaWAN base stations alongside Wi-Fi community networks to allow seamless data transfer and access [16]. In the United States, Amazon is deploying LoRa for its Sidewalk networking platform, which has been likened to a crowdsourced wireless network for neighborhoods [19]. Semtech has also launched its YoSmart and YoLink platforms to allow home connectivity using LoRa-based smart hubs, with up to a quarter-mile radius [20]. Collectively, the smart home market is expected to reach nearly US $155 billion by 2023, with the United States accounting for 40% of this, ensuring a rich innovation pipeline for subsequent global adoption [21]. Given that health care costs represent considerable proportions of gross domestic product (United States 16.9%, Switzerland 12%, sub-Saharan Africa 6.1% [ranging from 2.14%-16.1%], China 5%, India 3.6%, Organisation for Economic Co-operation and Development [OECD] average 8.8%), there is natural interest in deployment of these technologies as a component of digital health [22].

## IoMT Devices

Growth in wireless and mobile technologies has been particularly strong in the health sector, establishing a new category denoted the IoMT [23]. By current estimates, there are more than 3.7 million IoMT devices in use currently and a report by Allied Market Research forecasts IoMT within the health care market will grow to US $332 billion by 2027 [24]. This spans a range of handheld and body-worn devices (smart pad, smartphone, wrist-worn trackers, smart rings), wireless sleep monitors, smart refrigerators (which monitor dietary intake), and voice analyzers capable of diagnostic interrogation (eg, Alexa, Siri). It is easy to see how communication with residential IoMT devices can fuel digital health applications given that many individuals spend the majority of the day at home (and often almost the entire day during the COVID-19 pandemic). Rich composite data streams are available and could be useful for addressing disease-specific parameters (eg, diet and exercise activity for cardiometabolic illness, or voice intonation and social network activity in depressive disorders) and also for providing interventions (eg, reordering prescription medications, voice- or video-guided instructions for use of medicaments). Driving this growth are new technologies that are ever more powerful, miniaturized, and affordable. For example, tags (using near-field communication or radio frequency identification technology) as small as 1 mm^3^ can now be molded into drug packaging during manufacture, allowing patients to track delivery and access information from their smartphone [25]. Bluetooth modules mere millimeters square with shelf lives of many years can be added to devices and activated by a user—for example, on a smart pack to confirm a drug dose has been administered [26]. The deployment of these and “ready to connect” devices that have eSIMS integrated for over-air activation promises to greatly aid our efforts to monitor the health care supply chain [27], counterfeiting and security, and drug and diagnostics adherence rates. Critically for individuals in developing nations, many of these options do not require the use of a mobile phone. Direct-to-cloud capabilities are available in many areas, and combinations of fog and edge computing (allowing local processing of data prior to pushing to the cloud) are available in others [28]. Additionally, voice messaging from devices can be activated by the recipient of a package, requiring only simple microprocessors coupled to a piezo speaker and powered by a button cell. There even exists the option to record a simple return message onto such a device, which would then be transmitted to a base station (eg, via a LoRa network in small data packets). Such “return to sender” capabilities in essence close the loop between the patient and health care provider, confirming the participatory component of P4 medicine (predictive, preventive, personalized, and participatory) [29]. Ultimately, outcome measures will dictate the degree to which such connectivity will be adopted. However, early research on adherence rate improvements among patients using smart connected autoinjectors bodes well for deployment [30].

## Use Cases of BTM

Myriad exciting opportunities present themselves for BTM technology to enhance patient care and advance managed health care at the community level. From the drug product side, the ability to track supplies down to the individual package level will allow deployment of state-of-the-art logistics approaches developed in the online retail industry. Receipt by the patient at home, at a pharmacy, or care facility is possible, independent of how it was transported (truck, mail, parcel pod, drone, etc). This can also serve as an anticounterfeiting step, and ensure medications do not get into the wrong hands. Patient adherence can be monitored by dose verification technology (eg, smart blister pack, smart pill bottle, smart autoinjector) and supplies can be reordered to ensure no interruption. Where relevant, patient training could be offered via video/voice recording, connection to a smart television, or voice commander in the home. This could also lend itself to community-based medicine through peer groups. For example, smart grids around senior living communities could facilitate group support interactions through communication with smart packaging. Such has been seen to have a positive impact on outcome measures—for example, cardiovascular disease management through group exercise routines among peers [31]. Such can promote a wellness-oriented mindset, an important behavioral driver for long-term impact. With entire populations rendered housebound due to the COVID-19 pandemic and recreational facilities closed, the notions of community wellness and maintaining physical activity have seen added interest and are likely to stay beyond the pandemic.

## Deployment for Global Health

Use in developing nations, particularly rural areas where broadband networks are often unavailable, would seem an attractive proposition for use of BTM IoMT on unlicensed LPWANs. Indeed, such approaches have already been piloted in regions of sub-Saharan Africa, with successful outcomes reported [32]. Despite this validation, many studies have remained at the concept/pilot stage due to a lack of infrastructure for implementation, leading to a degree of frustration, which should be addressed as a matter of priority [32]. Pressure continues to mount, as evidenced by activities among several African nations to address mental health using mobile health tools during the COVID-19 crisis [33].

One concrete example of success was the SIMpill program trialed in South Africa. The system uses a smart drug container that sends a wireless signal when the cap is removed, signaling an adherence-related action by the patient [34]. Increased patient adherence rates ranging from 22% up to 90% were observed, suggesting fertile ground for widespread deployment [34].

In a recent study in Malawi, care for women with HIV was assessed using various technologies with a view to maximizing adherence for patients prescribed antiretroviral medications [35]. The study focused on evaluating 3 technologies for monitoring and supporting engagement in HIV care and the security screens used to validate patients’ identities. Although SMS text messaging and SIM card screening were viable, biometric-based (fingerprints) ID verification showed the highest level of engagement. Drivers cited included eliminating financial barriers (costs of phone and service), security concerns (third parties gaining access to device), consistency (trading of phones/cards), and lack of device literacy [35].

## Supply Chains and Clinical Trials

The studies in Malawi hint at the prospect that rural (and urban) drug dispensaries of the future might possibly function through IoMT, allowing patient verification by biometric scan through an LPWAN (or opening of smart pack/parcel pack medications delivered by drones). There seems little doubt that the technology can assist in the tracking of drug supply from manufacturer to patient and verifying adherence. The key to improved patient outcome measures lies in frequency of data capture and this should create minimal friction for the patient. As we have learned from longitudinal studies at scale, where we live affects our health and the ability to secure rich data over time is one of the keys to success in the field [36-38].

These same approaches would also benefit clinical trials being conducted in remote regions, where patient tracking and monitoring are pivotal components of trial design. Modern approaches often dictate highly segmented trials and the ability to effectively reach, engage, and monitor subpopulations is critical to addressing health disparities over time by facilitating trials with sufficient power [39].

## Emergency Management

Additional opportunities abound in the form of emergency alert systems for patients. For example, lack of communications infrastructure can place pregnant mothers in a vulnerable situation in rural areas. As has been noted, makeshift communication methods used by patients in developing nations range from use of carrier pigeons to alerting passing motorists of the need for assistance, with delays leading to fatalities [40]. LPWAN could address this need and—combined with active monitoring—be used to establish a form of emergency service channel as is the case in the United States with citizens band radio channels 9 (land-based) and 16 (marine). Such might become a near-term goal for collaborations between the World Health Organization and its member nations.

There have been additional developments in this regard from the Google X team and their “Project Loon,” which is developing balloons that are deployed in the stratosphere to provide wireless connectivity in remote areas around the globe. Though a current limiting factor is the 100-day service life of the balloons, they have already been deployed to assist populations in Peru impacted by floods and over Puerto Rico following Hurricane Maria, and have recently been deployed in parts of Africa [41].

## Conclusion and Next Steps

The era of connected health is upon us, and the range of possibilities for deployment of IoMT devices is growing apace. Near-term expected developments that will impact global health are on the horizon. The ability to track medication shipment, delivery, and activation through smart packaging merely awaits coordination and scale.

For any systems to embed into standard practice in managed health care, they will need to be mindful of human behaviors and align with motivational factors. Groups addressing human factors engineering and studying real-world evidence will play leading roles in this, guided by insights from behavioral psychology including the models outlined by Fogg [42], Maslow [43], and Russell [44].

One can foresee devices that capture and display information changing from merely having a reporting role to having a predictive one with the benefits of artificial intelligence and machine learning trained to the user/patient. Whether data are displayed on a handheld device, through smart glasses, or on surfaces holographically [45], the utility of the data relies on contextual and relational awareness requiring careful design.

One of the early hopes for connected health in the pharmaceutical industry was the concept of the “smart pill,” which could be tracked as it progressed through the gastrointestinal tract to confirm patient medication adherence. Though this remains elusive as a marketed product [46], future developments could be anticipated—for example, highly miniaturized transmitters activated when the drug encounters the low pH of the stomach or the higher pH of the ileum, sending a wireless signature directly to the cloud (or to mist or LPWAN), without the need for a smartphone [47].

## Remote Health Monitoring

In addition to confirming medication dosing wirelessly, our effluent streams could become sources for rich connected health information. There is an increasing awareness of the importance of the microbiome and its implications for health and P4 medicine [48]. To capitalize on this knowledge, a properly designed “smart toilet” could analyze and track components tied to the individual (eg, as a wireless detector for the aforementioned smart pill components postactivation, or as electrochemical sensors that detect microbiota-related degradation products). There are also a number of urinalysis diagnostic kits available and FDA approved that provide colorimetric analysis for biomarker levels in effluent streams [49]. Examples include metabolites tied to diseased states, with indicator cards imaged using a smartphone and uploaded to the cloud and the diagnostics provider [50]. Such analytical systems could presumably be embedded to allow direct to cloud services from toilets, and could be demonstrated in a use case at assisted living centers. Additional rationale for this approach stems from very recent screening of effluents by regional health authorities to track SARS-CoV-2 viral spread [51].

Another possibility of direct to cloud services could be systems modeled after unmanned pop-up health clinics. Originally trialed in the United States [52,53], they have become widely adopted in China, where they were introduced to address the scale of services needed in heavily populated urban areas [54]. Biometric verification at the patient level could allow access to services through LPWAN—for example, dispensing medications, or depositing a biological sample that does not require blood draw (sweat, tears, saliva). The facility could then analyze data locally and upload to the cloud, alerting health care providers if the need for intervention arises, or confirming medication adherence for payers.

**Figure 3:**
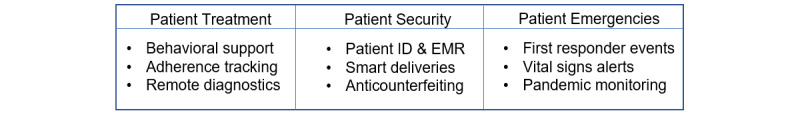
Potential utilization of Beyond the Mobile technologies in global health care. EMR: electronic medical record.

Such systems would also prove useful in the identification, tracking, and control of pandemics [51,55]. Both HIV and COVID-19 have had a severe impact in developing nations and such systems may offer a cost-effective method to provide needed services in remote areas [56]. It should also be noted that BTM applications will likely develop alongside current digital health technologies and the many groundbreaking benefits they have provided for chronic care and emergency management [57]. The possibilities are wide ranging (Figure 3) and it will be incumbent on the global health community to create solutions that will have lasting impact over time. *Think locally* *but* *act globally* may become a mantra for this challenge.

## Abbreviations

BTM: Beyond the Mobile
IoMT: Internet of Medical Things
LoRaWAN: long-range wide area network
LPWAN: low-power wide area network
